# Validation of a microwave energy meter to non-lethally estimate energetic reserves in adult sturgeon

**DOI:** 10.1093/conphys/coad023

**Published:** 2023-05-09

**Authors:** Nicole J Daigle, Matea A Djokic, Kevin M Kappenman, T Gibson Gaylord, Sierra Quinn, Christine E Verhille

**Affiliations:** Department of Biology, University of New Brunswick, Fredericton, New Brunswick, E3B 5A3, Canada; Department of Ecology and Evolutionary Biology, University of California, Irvine, CA, 92697-2525, USA; Bozeman Fish Technology Center, U.S. Fish and Wildlife Service, Bozeman, MT, 59715, USA; Bozeman Fish Technology Center, U.S. Fish and Wildlife Service, Bozeman, MT, 59715, USA; Department of Ecology, Montana State University, Bozeman, MT, 59715, USA; Department of Ecology, Montana State University, Bozeman, MT, 59715, USA

**Keywords:** sturgeon, management, energetics

## Abstract

Whole-body (WB) energetic reserves influence fish survival, growth, and reproduction but are typically quantified using lethal methods (*i.e.* proximate analyses) or interpreted through body condition indices. Energetic reserves can impact population dynamics through influences on growth rates, age-at-first-reproductive-maturity, and spawning periodicity at the individual-fish level, especially in long-lived sturgeon species. Therefore, a non-lethal tool to track the energetic reserves of endangered sturgeon populations could inform adaptive management and further our understanding of the sturgeon’s biology. The Distell Fatmeter is a microwave energy meter that has been validated to non-lethally estimate energetic reserves in some fish species, but never successfully for sturgeon. Here, stepwise linear regressions were applied to test commonly monitored body metrics and Fatmeter measurements at nine different anatomical sites on captive adult pallid sturgeon (*Scaphirhynchus albus*; total length of 790–1015 mm; WB lipid of 13.9–33.3%) compared with WB lipid and energy content determined by proximate analyses. Fatmeter measurements alone explained approximately 70% of the variation in WB energetic reserves, which outperformed models considering body metrics alone by a margin of approximately 20%. The top-ranked models based on AICc score (second-order Akaike Information Criterion) included a combination of body metrics and Fatmeter measurements and accounted for up to 76% of the variation in WB lipid and energy. We recommend the incorporation of Fatmeter measurements at a single site located dorsally to the lateral scutes at the posterior end of the fish above the pelvic fins (U-P) into conservation monitoring programs for adult pallid sturgeon (total length [TL] ≥ 790 mm; fork length [FL] ≥ 715 mm) and the cautious application of Fatmeter measurements for sturgeon between 435 and 790 mm TL (375–715 mm FL). Measurements at this U-P site combined with body mass explained approximately 75% of the variation in WB lipid and energy.

## Teaser

We successfully validated the Fatmeter as a tool to obtain precise, non-lethal estimates of whole-body energetic reserves in captive adult pallid sturgeon. We recommend the implementation of Fatmeter measurements in monitoring programs for free-living sturgeon and include measurement sites and formulae to estimate whole-body lipid and energy using raw measurements.

## Introduction

Whole-body (WB) energetic reserves have important influences on physiological processes governing survival, growth, and reproduction (Kooijman, 2009; Deslauriers *et al.,* 2016). Shortages of energetic reserves can constrain survival (Randall *et al.,* 2017; Steffensen, 2018), metabolism (Daigle *et al.,* 2021), and reproduction (Barneche *et al.,* 2018) across a large array of fish taxa. WB energetic reserves are typically determined through lethal methods (*i.e.* proximate analyses); however, this is not practical for sturgeon species because most are endangered or of conservation concern (IUCN, 2022). Reliable, non-lethal tracking of fluctuations in energetic reserves could be informative to adaptive management and improve our general understanding of the sturgeon’s biology.

The pallid sturgeon (*Scaphirhynchus albus*) is a long-lived, slow-growing, and intermittently spawning fish species that was listed as endangered in 1990 due to population declines and little to no natural recruitment (USFWS, 1990; Fuller *et al.,* 2007; Jordan *et al.,* 2016; Holmquist *et al.,* 2019). Successful management interventions have slowed their decline, but many key aspects of pallid sturgeon biology remain elusive, preventing an accurate evaluation of how this species may respond to management actions. For example, through the Pallid Sturgeon Conservation Augmentation Program, hatchery propagation and stocking of pallid sturgeon began in the 1990s (USFWS, 2014; USFWS, 2019) and has achieved high survival rates (Rotella, 2017). However, growth rates for recently stocked cohorts relative to the earliest stocked cohorts have slowed (Wilson *et al.,* 2017; Steffensen *et al.,* 2019). In addition, the mechanisms underlying the variation in size- and age-at-first-maturity among pallid sturgeon populations inhabiting the Missouri and Yellowstone Rivers above Fort Peck Reservoir, the Missouri River below Fort Peck, and captive pallid sturgeon are not fully understood (Hamel *et al.,* 2014; Jordan *et al.,* 2016; Holmquist *et al.,* 2019; Hamel *et al.,* 2020; Cox *et al.,* 2022) and is likely related to the maintenance and accumulation of WB energetic reserves. Furthermore, pallid sturgeon relative condition (Kn; the ratio of the observed body mass [BM] to the length-based predicted BM for a fish) has been declining since 2004, and from 2012 to 2015, a small number of extremely emaciated pallid sturgeon were observed in the lower Missouri River (Steffensen and Mestl, 2016; Steffensen, 2018).

To date, the monitoring of free-living pallid sturgeon WB energetic reserves has been limited to Kn calculations based on body metric measurements. Relative condition is often considered an indicator of relative energy stores within fishes (Blackwell *et al.,* 2000; Wuenschel *et al.,* 2019). Previous studies have shown Kn for reproductive pallid sturgeon to be greater than for non-reproductive sturgeon of the same sex and fork length (FL; Steffensen and Mestl, 2016). Although Kn can be a valuable metric to assess sturgeon body condition, it does not correlate well with WB energetic reserves in captive juvenile pallid sturgeon (Meyer *et al.,* 2016; Djokic *et al.,* 2022). Given the long lifespan (Braaten *et al.,* 2015), high survival rates after age two (Rotella, 2017), and influences of WB energetic reserves on reproduction (Blackwell *et al.,* 2000), non-lethal and precise monitoring of WB energetic reserves can provide an important improvement in monitoring the status of free-living populations of pallid sturgeon, to better understand their biology and implement adaptive management.

The Fatmeter measures microwave energy transmission to estimate the crude fat content of fish based on the relationship between body lipids and moisture (Distell, 2011; Lee *et al.,* 2016 and sources cited within), and it has been proposed as a tool for estimating WB energetic reserves in fish. Measurements performed with the Fatmeter were validated as an estimate of WB energetic reserves in Atlantic herring (*Clupea harengus harengus*; Vogt *et al.,* 2002), Pacific salmon (*Oncorhynchus spp*.; Colt and Shearer, 2001; Crossin and Hinch, 2005; Kaga *et al.,* 2009), channel catfish (*Ictalurus punctatus*; Mesa and Rose, 2015), Atlantic salmon (*Salmo salar*; Hendry and Beall, 2004), American shad (*Alosa sapidissima*; Bayse *et al.,* 2018), and Atlantic croaker (*Micropogonias undulatus*; Schloesser and Fabrizio, 2017) (see Supplementary Table S1); further, Distell has validated the Fatmeter for a substantial number of other fish species via in-house calibrations (Distell, 2011). However, Fatmeter measurements were not effective at estimating WB energetic reserves for striped bass (*Morone saxatilis*; Schloesser and Fabrizio, 2017), summer flounder (*Paralichthys dentatus*; Schloesser and Fabrizio, 2017), smallmouth bass (*Micropterus dolomieu;*Mesa and Rose, 2015), and walleye (*Sander vitreus*; Mesa and Rose, 2015). For juvenile pallid sturgeon, Fatmeter measurements alone were not effective at estimating WB energetic reserves but the combination of Fatmeter measurements with body metrics accounted for 40–45% of the variation in WB energetic reserves (Djokic *et al.,* 2022). It was hypothesized that the limited effectiveness of Fatmeter measurements to estimate juvenile pallid sturgeon WB energetic reserves was due to the small size of the sturgeon (≤480 mm total length [TL]), and a follow-up study on larger adult pallid sturgeon was recommended.

The objective of the current study was to measure the accuracy of the Distell Fatmeter (FFM-992, Distell, West Lothian, UK) independently and in combination with body metrics to estimate WB energetic reserves (WB lipid and energy) in captive adult pallid sturgeon. A secondary objective was to recommend an approach to implement Fatmeter measurements in monitoring programs for free-living pallid sturgeon, including measurement sites and formulae to convert raw site-specific Fatmeter measurements into estimates of WB lipid and energy.

## Materials and Methods

The sturgeon used for Fatmeter testing were the progeny of spawning events that took place between 2004 and 2005 at the Gavins Point National Fish Hatchery (NFH) in Yankton, SD. During the spawning events, multiple family crosses were done between 8 females and 15 males, yielding a total of 17 unique family crosses. Between July 8 and 11, 2019, 45 of these fish (now aged 14–15 years) were transported to the Bozeman Fish Technology Center, BFTC, in Bozeman, MT, and left to acclimate for 4 months. From spawning through the first 4 months at BFTC, the fish were reared within the parameters recommended by the Upper Basin Pallid Sturgeon Propagation Committee (2005). Methods were approved by the Institutional Animal Care and Use Committee (IACUC) to ensure animal welfare was maintained at all times.

Before sampling to test the efficacy of Fatmeter measurements to estimate WB energetic reserves, feed rations were manipulated for 4.5 months to promote variation in WB energetic reserves within the study population. On November 4, 2019, the fish were measured for BM (g) and TL (mm), then they were randomly separated across nine tanks (five fish per tank) with three replicate tanks for each of the three feed ration (treatment) groups: high feed (0.75 ± 0.04% tank BM/day; mean ± standard error [SE]), medium feed (0.29 ± 0.05% tank BM/day) and fasting (0 ± 0% tank BM/day). The fish were fed their respective rations daily (excluding weekends) with 6-mm sinking pellets of a high-protein diet (Classic Trout Skretting, Tooele, UT; 40% protein, 12% oil, 9% moisture, 3% fibre, 12% ash and 14.6 MJ/kg digestible energy), delivered via belt feeder. The type of feed used was in accordance with the commercial diet recommendations from the Upper Basin Pallid Sturgeon Propagation Committee (2005). All tanks were held at a constant temperature of 17.8 ± 0.02°C (mean ± SE; min: 15.8°C; max: 19.2°C), and dissolved oxygen (DO) saturation was maintained at >80%. Temperature and DO saturation were monitored using temperature loggers (HOBO MX2201, Onset, MA) and an oxygen meter (Pro 2030, YSI, OH).

Fish inventories were completed monthly to adjust feeding with changes in tank biomass and to visually monitor general health, with a final (lethal) inventory at the end of the manipulation. During inventories, the fish were briefly removed from their tanks to check for any visual signs of illness (*e.g.* body and gill colouration or the appearance of surface lesions or sores) and to measure their BM and TL. Relative condition (Kn; no units) was calculated as the ratio of observed BM to expected BM. Expected BM was calculated separately based on mass–length relationships derived for the entire Missouri River (basin-wide) pallid sturgeon population (Shuman *et al.,* 2011) and the pallid sturgeon population inhabiting the Missouri River downstream of the Fort Peck Dam (Randall *et al.,* 2017) as detailed below. Fork length was estimated from observed TL using a species-specific length conversion (Keenlyne and Maxwell, 1993). The Kn of the fish was calculated separately based on Shuman *et al.*’s (2011) expected mass (KnS) and Randall *et al.*’s (2017) expected mass (KnR):

[1] Log_10_ (BM) = −6.2561 + 3.2932 × Log_10_ (FL) (Shuman *et al.,* 2011).

[2] Log_10_ (BM) = −5.9205 + 3.1574 × Log_10_ (FL) (Randall *et al.,* 2017).

At the end of the 4.5-month feed manipulation (March 18, 2020), the fish were euthanized via anaesthetic overdose of 250 mg/L of tricaine methanesulfonate, MS-222 (Syndel, WA), buffered in a 1:2 ratio of MS-222 to sodium bicarbonate for Fatmeter and body metric measurements, sex and gonad developmental stage determination, and for lethal proximate determination of WB energetic reserves. Notably, before the fish were exposed to the anaesthetic, a blood sample (≤3 ml) was taken for a companion study (data not included) and was not expected to influence Fatmeter measurements or analytical quantification of WB energetic reserves. Immediately after removal from the anaesthetic, Fatmeter, TL, and BM measurements were performed. The carcasses were then frozen and stored at −20°C until later sex and gonad stage assessment and preparation for proximate analyses.

**Figure 1:**
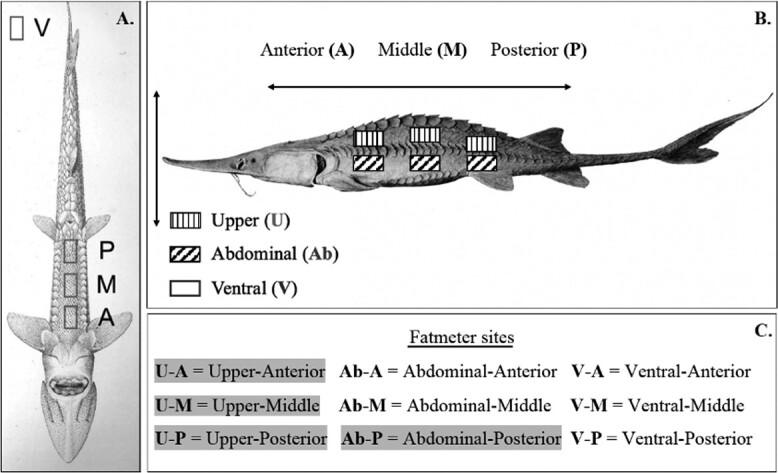
Visual representation of the ventral (panel A) and lateral (panel B) Fatmeter measurement sites (shown by patterned rectangles) that were measured to estimate whole-body lipid and energy in captive adult pallid sturgeon, *Scaphirhynchus albus*. The sites highlighted in grey were included in the analyses. The Fatmeter site codes (panel C) denote the site’s position on the dorsoventral (U: upper, Ab: abdominal, V: ventral) and anteroposterior (A: anterior, M: middle, P: posterior) axes. This figure uses modified images of a shovelnose sturgeon, *Scaphirhynchus platorynchus* (A; Heckel, 1836) and a white sturgeon, *Acipenser transmontanus* (B; Forbes and Richardson, 1908).

Nine non-overlapping Fatmeter sites were measured in this study: Upper-Anterior (U-A), Upper-Middle (U-M), Upper-Posterior (U-P), Abdominal-Anterior (Ab-A), Abdominal-Middle (Ab-M), Abdominal-Posterior (Ab-P), Ventral-Anterior (V-A), Ventral-Middle (V-M), and Ventral-Posterior (V-P) (Figure 1). All Fatmeter measurements were taken using the Distell Fatmeter on the RESEARCH-1 setting. This setting specified duplicate uncalibrated measurements at a single site and output the average. The upper (U-) and abdominal (Ab-) Fatmeter sites used in the current study were selected based on a preliminary analysis that assessed the coefficient of variation (CV) of triplicate site measurements on a population of juvenile and adult pallid sturgeon that were not included in our study population (data not shown). As a result of this analysis, the Ab-A and Ab-M sites were excluded from the analyses due to an average CV ≥ 0.15. The three ventral Fatmeter sites (V-A, V-M, and V-P) were not included in the preliminary analysis but were also measured during the lethal inventory of the sturgeon.

Sex and gonad developmental stages were determined via visual examination of the gonads. The gonads of all fish were relatively thick allowing male and female sturgeon to be distinguished based on the presence of oocytes (Webb *et al.,* 2019). The female gonad developmental stage was classified as earlier or later than the late vitellogenic developmental stage based on the darkening of the ovarian follicles (Webb *et al.,* 2019). Males were not classified into developmental stages because their developmental stages cannot be confidently assigned through visual inspection alone (Webb *et al.,* 2019).

Whole-body energetic reserves were determined through proximate analyses of subsamples of each sturgeon’s homogenized ground carcass, performed by IEH Laboratories (Greeley, CO, USA). Each entire frozen sturgeon carcass was thawed and then homogenized in an industrial meat grinder (Hobart Corporation, Troy, OH, USA) at the BFTC. Frozen 100-g subsamples of each ground carcass were shipped to IEH Laboratories for duplicate determination of WB lipid and energy content. WB lipid (%) was determined following the ether extraction method (AOAC analysis method 960.39), and WB energy (kcal/100 g) was calculated using the Atwater factors equation (AOAC, 2012):

[3] WB energy (kcal/100 g) = (9 × WB lipid) + (4 × protein) + (4 × carbohydrates).

All analyses were completed in R, version 4.2.2 (R Core Team, 2023). The raw data were visualized as plots of WB lipid and energy regressed onto body metrics and all combinations of Fatmeter sites to identify potential candidate explanatory variables to estimate WB lipid or energy. A Rosner test (Rosner, 1983) was first used to flag and remove outliers in the dataset; it was subsequently run a second time to ensure no outliers remained. A Shapiro–Wilk test was then used to assess the distributions of all parameters: WB lipid and energy were flagged as non-normal and subsequently transformed to normal distributions using power (WB Lipid^0.25^) and negative reciprocal power (−WB Energy^-0.7^) transformations, respectively, following the ladder of powers (Velleman and Hoaglin, 1981). According to a Levene test, all parameters displayed homogeneity of variance. Lastly, variance inflation factors (VIF) were calculated on all possible combinations of explanatory variables using the “vif” function from the car package (Fox *et al.,* 2021). A cut-off value of 5 was applied to the VIF scores to indicate multicollinearity; pairs of explanatory variables exceeding this cut-off were not included together in any subsequent models (Craney and Surles, 2002).

After assumption testing, linear regressions were used to determine if Fatmeter readings alone or in combination with BM, TL, and/or Kn (S or R) could effectively estimate the WB energetic reserves of the adult pallid sturgeon. A forward-selection regression (linear model) was run using the “lm” function in R to rank, according to AICc scores (a version of the Akaike Information Criterion that applies a correction for small sample sizes; Burnham and Anderson, 2004), all potential combinations of explanatory variables to explain variation in WB lipid and energy. Potential explanatory variables included all possible combinations of individual Fatmeter sites (averaged, as done in Crossin and Hinch, 2005 with Pacific salmon and Djokic *et al.,* 2022 with juvenile pallid sturgeon) and body metrics (BM, TL, KnS and KnR). In the first step of the regression, the best fit model (only one explanatory variable) was selected as the model with the lowest AICc score. The model was then iteratively expanded one new variable at a time to identify the best fit model at each iteration until the ΔAICc score between the penultimate and ultimate model differed by <2 (Burnham and Anderson, 2002).

The best fit models (based on AICc scores) and the recommended models (based on a combination of AICc scores and effective implementation of measurements in the field) were identified for WB lipid and energy. All competitive models, identified as the best fit models based on AICc scores, were considered when determining the AICc top-ranked and recommended models. The recommended models were determined based on the trade-off between statistical ranking (AICc score) and minimization of Fatmeter measurement sites to minimize fish handling time.

The influence of sex and gonad developmental stage on WB energetic reserves and the recommended models estimating WB energetic reserves were tested post-model selection for the recommended linear models including Fatmeter measurements and body metrics. For females only, *t* tests were used to test for differences in WB lipid or energy values between fish classified into pre- and post-late vitellogenic gonad developmental stages using the base R function “t.test” (Student *t* test). Differences in WB lipid or energy values between males and females were also tested using *t* tests. In addition, the recommended models resulting from the forward-selection regressions were re-run with sex added as an independent variable to test whether sex was a significant variable within the model; the gonad developmental stage was not tested as an independent variable with the recommended models because gonad developmental stage could only be determined for females.

## Results

On average, it took approximately 9 min to complete all Fatmeter site measurements (approximately 1 min/site). All ventral sites were eliminated from further consideration in the analyses due to inconsistent duplicate Fatmeter measurements noted during inventories. Consequently, the analyses included four Fatmeter sites (U-A, U-M, U-P, and Ab-P; Figure 1),

At the final (lethal) inventory, the mean BM and TL of the 45 experimental fish were 3306.7 ± 118.5 g (mean ± SE; min: 1626 g; max: 5429 g) and 912.5 ± 8.2 mm (mean ± SE; min: 764 mm; max: 1015 mm), respectively. Three fish were prematurely euthanized during the study due to severe illness or injury noted during the inventories between January 20 and February 24, 2020. Because the objective was to evaluate Fatmeter measurements for monitoring free-living adult pallid sturgeon (that could exhibit illness), the two ill experimental fish were included in the analyses. The third fish (from the high-feed group) was excluded from the analyses due to an injury of the caudal fin that made measures of TL and Kn unreliable. An additional fish (from the medium-feed group) was identified as an outlier via the Rosner test and was removed from the analyses after further inspection of the raw data (WB lipid measurements were below the detectable limit from IEH laboratories, <1.3%). Therefore, we had a total of 43 sturgeon with an average BM of 3342.7 ± 121.2 g (mean ± SE; min: 1626 g; max: 5429 g) and TL of 915.8 ± 7.8 mm (mean ± SE; min: 790 mm; max: 1015 mm). As anticipated, the study population displayed a wide range of WB lipid (13.9–33.3%), and energy (187.0–351.5 kcal/100 g) content (Table 1).

**Table 1:** Descriptive statistics of whole-body proximate analyses, body mass (BM), total length (TL) and relative condition (Kn; based on Randall’s [KnR] and Shuman’s [KnS] FL-at-BM relationship) for captive adult pallid sturgeon, *Scaphirhynchus albus*. For the determination of Kn, TL observations were converted to fork length (FL) as per Keenlyne and Maxwell (1993). SE represents standard error, n = 43

Parameter	Mean	SE	Minimum	Maximum
Lipid (%)	20.9	0.7	13.9	33.3
Energy (kcal/100 g)	245.2	6.3	187.0	351.5
Ash (%)	2.5	0.1	1.8	3.3
Carbohydrates (%)	1.0	0.2	0	4.9
Moisture (%)	62.2	0.7	52.0	69.1
Protein (%)	14.3	0.2	12.3	16.5
BM (g)	3342.7	121.2	1626.0	5429.0
TL (mm)	915.8	7.8	790.0	1015.0
KnR (no units)	1.6	<0.1	1.3	2.3
KnS (no units)	1.4	<0.1	1.1	2.0

The model summaries (AICc, *P* value, and R^2^) for the top-ranked competitive models identified via the forward-selection regressions are presented in Table S2 (see Supplementary Data), in which WB lipid and energy were separately regressed onto body metrics only, Fatmeter measurements only, and a combination of the two. The AICc top-ranked models included Fatmeter measurements and body metrics and explained ≥25% more of the variation in WB energetic reserves than the best fit models considering only body metrics as explanatory variables (Table 2). The best fit models considering only body metrics included KnR as the single explanatory variable and explained 49 and 50% of the variation in WB lipid and energy, respectively. When considering only Fatmeter sites as explanatory variables, the best fit model was Avg(Ab-P, U-A, U-P) for WB lipid (R^2^ = 0.71) and Avg(Ab-P, U-M, U-P) for WB energy (R^2^ = 0.72). Therefore, Fatmeter measurements alone explained approximately 20% more of the variation in WB energetic reserves than models including only body metrics. The best fit models overall were those that combined body metrics and Fatmeter measurements, with Avg(Ab-P, U-A, U-P) and BM as the AICc top-ranked model for WB lipid (R^2^ = 0.76) and U-P and BM as the AICc top-ranked model for WB energy (R^2^ = 0.76).

**Table 2:** Regression models (with coefficients) for estimating the whole-body (WB) lipid (%) and energy content (kcal/100 g) of captive adult pallid sturgeon, *Scaphirhynchus albus* (body mass [BM] of 3342.7 ± 121.2 g, mean ± SE), n = 43. The recommended models are presented in comparison with the best fit models using only body metrics and the AICc top-ranked models. The recommended models were determined based on the trade-off between AICc ranking and the minimization of the number of Fatmeter measurements to minimize fish handling time. All *P* values were <0.001. Abbreviations: AICc is second-order Akaike Information Criterion; KnR is Randall’s relative condition (no units; based on Randall’s FL-at-BM relationship); Avg denotes the average of the specified sites; The Fatmeter site codes denote the site’s position on the dorsoventral (U: upper, Ab: abdominal) and anteroposterior (A: anterior, M: middle, P: posterior) axes

Estimate	Model	AICc	R^2^
	*Body metrics only (best fit model)*		
WB lipid	(1.52 + [0.37 × KnR])^4^	−84.8	0.49
WB energy	{−1 ÷ ([−0.034] + [0.0076 × KnR])}^(10/7)^	−421.7	0.50
	*AICc top-ranked model*		
WB lipid	{1.38 + ([1.24 × 10^−2^] × Avg[Ab-P, U-A, U-P]) + ([4.07 × 10^−5^] × BM)}^4^	−115.0	0.76
WB energy	<−1 ÷ {[−3.68 × 10^−2^] + ([2.26 × 10^−4^] × U-P) + ([1.13 x 10^−6^] × BM)}>^(10/7)^	−452.0	0.76
	*Recommended model (Applicable to minimum size of 435–790 mm TL)*		
WB lipid	{1.37 + ([1.14 × 10^−2^] × U-P) + ([5.30 × 10^−5^] × BM)}^4^	−114.5	0.75
WB energy	<−1 ÷ {[−3.68 × 10^−2^] + ([2.26 × 10^−4^] × U-P) + ([1.13 × 10^−6^] × BM)}>^(10/7)^	−452.0	0.76

**Figure 2:**
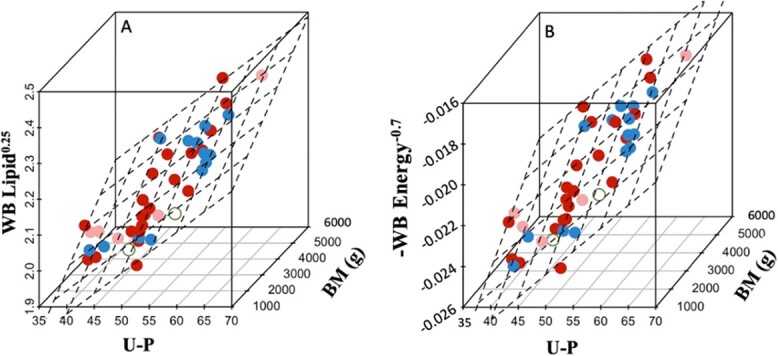
The recommended models for estimating whole-body (WB) lipid (panel A) and WB energy (panel B) of captive adult pallid sturgeon (BM of 3342.7 ± 121.2 g, mean ± SE), n = 43. Values for WB lipid and energy were transformed (WB Lipid^0.25^ and −WB Energy^-0.7^); the units for the pre-transformed values are % for WB lipid and kcal/100 g for WB energy. The abbreviation U-P denotes the Upper-Posterior Fatmeter site. Data point colour and shade, respectively, denote sex (red = female; blue = male, unfilled = sex undetermined) and, for females only, gonad developmental stage (dark red = post-late vitellogenic; light red = pre-late vitellogenic).

In the selection of the final recommended models, we considered the trade-off between the model fit and the minimization of fish handling time (*i.e.* one Fatmeter measurement site). Consequently, the AICc top-ranked models were compared with the best fit models including Fatmeter measurements at only one site. The model including U-P and BM was the best fit single Fatmeter site model for both WB lipid (R^2^ = 0.75) and energy (R^2^ = 0.76; Table 2; Figure 2). These models explained similar amounts of variation and had ΔAICc scores <2 compared with the AICc top-ranked models.

Neither sex nor gonad developmental stage was significantly related to WB lipid or energy. Of the 43 fish included in model development, 22 were identified as male, 19 were identified as female, and two were of unidentified sex. Of the females, 14 were classified as the post-late vitellogenic stage and five were classified as the pre-late vitellogenic stage. All *t* tests showed WB lipid and energy to not differ significantly between males and females (*P* = 0.30 for WB lipid and *P* = 0.35 for WB energy) or between pre- and post-late vitellogenic females (*P* = 0.62 for WB lipid and *P* = 0.61 for WB energy). In addition, when sex was added as an independent variable to the recommended models estimating WB lipid and energy, the sex term was not significant (*P* = 0.54 for WB lipid and *P* = 0.36 for WB energy).

## Discussion

Here we present the first successful validation of a microwave energy meter, the Distell Fatmeter, to non-lethally estimate WB energetic reserves in an adult sturgeon species, after our previous attempt with juvenile pallid sturgeon (Djokic *et al.,* 2022). Because sturgeon species are long-lived periodic reproducers, spawning periodicity is thought to be intrinsically driven by the rate at which an individual sturgeon can accumulate lipid stores between successive spawning years (Kooijman, 2009). Pallid sturgeon life history includes a transition from an insectivorous juvenile stage to a piscivorous adult stage (Grohs *et al.,* 2009), with success at piscivory likely driving the accumulation of energetic reserves required to reach reproductive maturity (Holmquist *et al.,* 2019). Therefore, precise estimates of WB energetic reserves over time could provide important information on the impacts of management actions, fluctuations in prey species abundance (Gerrity *et al.,* 2006) or river conditions on reproduction and the subsequent population dynamics of pallid sturgeon and other sturgeon species. We provide recommendations for the implementation of Fatmeter measurements to obtain fast, non-lethal, and precise estimates of WB energetic reserves in wild-captured adult pallid sturgeon.

Attempts to achieve variation in Kn within the study population, which resembles the range occurring within free-living populations, were successful at the high extremes of KnR but fell short of reaching the low extremes observed in wild-captured adult pallid sturgeon. The 95% confidence intervals of wild-captured adult pallid sturgeon since 2003 vary from 0.90 to 1.10 (min: 0.31; max: 2.19; Randall *et al.,* 2017). In the current study, our maximum KnR (2.3) of captive-reared sturgeon corresponded with reported (wild-captured) KnR maximums; however, despite 4.5 months of fasting for one treatment (feed ration) group, the minimum KnR (1.3) achieved in the current study was much higher than reported KnR minimums and even exceeded the upper 95% confidence interval (Randall *et al.,* 2017). Despite the limited lower range of KnR achieved in the current study, the maximum observed WB lipid and energy values more than doubled the minimum, and the sturgeon with the highest BM was more than three times heavier than the smallest sturgeon. Although we were unable to test the Fatmeter on extremely emaciated adult pallid sturgeon, the strong relationship across a broad range of WB lipid (13.9–33.3%), energy (187–351.5 kcal/100 g), and KnR (1.1–2.3) demonstrates that the Fatmeter is a valuable tool for estimating WB energetic reserves in the population investigated in this study.

Fatmeter measurements reliably estimated WB energetic reserves in captive adult pallid sturgeon ≥790 mm TL. For models including only Kn, which is often interpreted as a proxy for energetic reserves (Blackwell *et al.,* 2000; Wuenschel *et al.,* 2019), KnR performed slightly better than KnS, but both indices explained approximately 50% of the variation in WB lipid and energy (Table 2; Supplementary Table S2). Fatmeter measurements alone explained 20% more of the variation in WB energetic reserves than models using only body metrics. The AICc top-ranked models, which combined Fatmeter measurements with body metrics, accounted for approximately 75% of the variation in WB energetic reserves, which outperformed current monitoring practices using Kn to estimate WB energetic reserves by a margin of ≥25%.

Based on AICc score rankings and the minimization of the number of Fatmeter site measurements, the U-P site was the top-performing Fatmeter measurement site for estimating WB lipid and energy. Although the top-ranked model for estimating WB lipid included measurements at three different Fatmeter sites, a simplified model including only one Fatmeter site (U-P) performed comparably with similar AICc and explained variation (R^2^). Furthermore, the top-ranked model estimating WB energy included the U-P site as the only Fatmeter site. Considering the increased fish handling time required to collect multiple Fatmeter site measurements from endangered wild-captured sturgeon, we recommend the application of the models estimating WB lipid and energy based only on the U-P Fatmeter site and BM (Table 2; Figure 2).

Fatmeter measurements as estimates of WB energetic reserves in adult pallid sturgeon achieved comparable R^2^ values to those reported in Fatmeter validation studies on other fish species (Supplementary Tables S1 and S2). Fatmeter measurements alone have performed variably across fish species, with the highest amount of variation in WB energetic reserves explained in sockeye salmon, *Oncorhynchus nerka* (93–94%; Crossin and Hinch, 2005) and the lowest explained in smallmouth bass (0.02%; Mesa and Rose, 2015), striped bass (0.02–0.22%; Schloesser and Fabrizio, 2017), and juvenile pallid sturgeon (0–0.23%; Djokic *et al.,* 2022). For juvenile pallid sturgeon, combining BM with Fatmeter measurements as explanatory variables nearly doubled the R^2^ (to 0.45). This still remained much lower than the R^2^ values reported in the current study, which also included Fatmeter and BM measurements as explanatory variables but achieved R^2^ values of approximately 0.75. As discussed in detail by Djokic *et al.* (2022), higher levels of WB lipid (maximum of 11.3% in Djokic *et al.,* 2022 versus a minimum of 13.9% here) and reduced interference of scutes on the larger body surface of adult pallid sturgeon likely explain the improved amount of variation in WB energetic reserves explained by Fatmeter measurements. In addition, the high amount of variation in WB energetic reserves accounted for by Fatmeter measurements alone in adult pallid sturgeon suggests that microwave signals are capable of penetrating through to the deep visceral lipid stores of sturgeon. Interestingly, the top-performing Fatmeter measurement site differed between juvenile and adult pallid sturgeon; whether considered alone or in combination with body metrics, the U-A Fatmeter site performed best in juveniles (Djokic *et al.,* 2022), whereas the U-P site performed best in adults. These life stage differences may reflect variation in anatomy or lipid distribution with age.

The question that our current and previous study does not resolve is: what is the minimum sturgeon size allowing for Fatmeter measurements to estimate WB energetic reserves? Our previous study on juvenile pallid sturgeon found body metrics combined with Fatmeter measurements to explain, at best, 45% of the variation in WB energetic reserves for fish up to 480 mm TL (Djokic *et al.,* 2022). Here, the variation in observed data around the recommended model fit for adult pallid sturgeon passed the homogeneity of variance test, suggesting that the smallest fish (790 mm TL) tested here were not approaching the minimum size limit for effective estimation of WB energetic reserves. Therefore, the minimum sturgeon size for effective estimation of WB energetic reserves based on the U-P site and BM lies somewhere between approximately 480 and 790 mm TL. To further investigate this minimum size limit, we investigated the residuals for WB energetic reserves for the largest fish (≥435 mm TL; ≥375 mm FL) from Djokic *et al.* (2022) estimated with our recommended models. Although the residuals for these smaller fish showed a bias toward underestimating WB energetic reserves with our recommended model, the residuals fell within the range observed across the larger adult fish within the current study (see Supplementary Figure S1). Consequently, we suggest that the model recommended here could be applied with caution to fish <790 mm TL to estimate WB energetic reserves but should not be applied to fish <435 mm TL.

As with any tool, it is important to recognize the limitations of Fatmeter measurements in estimating fish WB energetic reserves. The variation in the relationship between Fatmeter measurements and WB energetic reserves may be affected by extreme habitat conditions (*e.g.* temperature or salinity) due to the effects of temperature on microwave transmission (Distell, 2011) and salinity on body fluids (Haller *et al.,* 2015). In addition, although the moisture–lipid relationship Fatmeter estimates of WB energetic reserves depends on has been shown to persist across a large taxonomic breadth of fish species, environmental conditions, and physiological states (Powell *et al.,* 2010; Distell, 2011; Lee *et al.,* 2016 and sources cited within), a moribund fish could experience a homeostatic breakdown that disrupts the moisture–lipid relationship; therefore, Fatmeter measurements should be interpreted with caution in conjunction with the Kn for a fish exhibiting signs of morbidity.

The objective of this study was to quantify the performance of Fatmeter measurements to estimate the WB energetic reserves of adult pallid sturgeon and to subsequently recommend best practices for using Fatmeter measurements as an estimate of WB energetic reserves in adult pallid sturgeon. Our results were consistent with previous studies validating Fatmeter measurements on fishes as an effective, non-invasive estimate of WB energetic reserves. Although this study was not designed to test for sex and maturity effects, a preliminary analysis with low sample sizes found no effect of sex or female gonad developmental stage on our recommended models. We recommend the incorporation of Fatmeter measurements at the U-P site into monitoring programs for adult pallid sturgeon (TL ≥790 mm; ≥715 mm FL) and the cautious application for sturgeon between 435 and 790 mm TL (375–715 mm FL) to estimate WB lipid and energy of individual sturgeon using the recommended models described in Table 2 and visualized in Figure 2. Because energetic reserves influence population dynamics via effects on growth rates, age-at-first-reproductive-maturity, and spawning periodicity, the improved performance of Fatmeter measurements over typically monitored Kn for estimating WB energetic reserves will provide important information to guide adaptive management and expand our knowledge of sturgeon biology while adding minimal time (~1 min) to sturgeon monitoring protocols.

## Funding

This work was supported by a United States Army Corps of Engineers grant, a Western Area Power Administration grant, and Montana State University funds (C.E.V.).

## Data availability

Raw data have been included with the Supplementary Material.

## Author contributions

N.J.D.: Data curation, methodology, formal analysis, visualization, writing (original draft preparation, review, and editing). M.A.D.: Conceptualization, data collection, manuscript review. K.M.K.: Funding acquisition, resources, conceptualization, manuscript review. T.G.G.: Resources, conceptualization, manuscript review. S.Q.: Conceptualization, data collection. C.E.V.: Funding acquisition, resources, conceptualization, supervision, methodology, data collection, writing (review and editing).

## Supplementary Material

Web_Material_coad023

## References

[ref1] AOAC (2012) *Official Methods of Analysis of AOAC International*, 19th Edition. AOAC International, Gaithersburg, vol 1-2

[ref2] Barneche DR , Robertson DR, White CR, Marshall DJ (2018) Fish reproductive-energy output increases disproportionately with body size. Science 360: 642–645. 10.1126/science.aao6868. 29748282

[ref3] Bayse SM , Regish AM, McCormick SD (2018) Proximate composition, lipid utilization and validation of a non-lethal method to determine lipid content in migrating American shad *Alosa sapidissima*. J Fish Biol 92: 1832–1848. 10.1111/jfb.13624. 29603209

[ref4] Blackwell BG , Brown ML, Willis DW (2000) Relative weight (Wr) status and current use in fisheries assessment and management. Reviews Fish Sci 8: 1–44. 10.1080/10641260091129161.

[ref5] Braaten PJ , Campana SE, Fuller DB, Lott RD, Bruch RM, Jordan GR (2015) Age estimations of wild pallid sturgeon (*Scaphirhynchus albus*, Forbes & Richardson 1905) based on pectoral fin spines, otoliths and bomb radiocarbon: inferences on recruitment in the dam-fragmented Missouri River. J Appl Ichthyol 31: 821–829. 10.1111/jai.12873.

[ref6] Burnham KP , Anderson DR (2002) AIC differences, Δ_i_. In: Burnham KP, Anderson DR, eds. *Model Selection and Multimodel Inference: A Practical Information-Theoretic Approach*, Second Edition. Springer, New York, pp. 70–72

[ref7] Burnham KP , Anderson DR (2004) Multimodel inference: understanding AIC and BIC in model selection. Sociol Methods Res 33: 261–304. 10.1177/0049124104268644.

[ref8] Colt J , Shearer KD (2001) Evaluation of the Use of the Torry Fish Fatmeter to Non-Lethally Estimate Lipid in Adult Salmon. Report: U.S. Army Corps of Engineers, Seattle, pp. 1–70

[ref9] Cox TL , Guy CS, Holmquist LM, Webb MAH (2022) Reproductive indices and observations of mass ovarian follicular atresia in hatchery-origin pallid sturgeon. J Appl Ichthyol 38: 391–402. 10.1111/jai.14339.

[ref10] Craney TA , Surles JG (2002) Model-dependent variance inflation factor cutoff values. Qual Eng 14: 391–403. 10.1081/QEN-120001878.

[ref11] Crossin GT , Hinch SG (2005) A nonlethal, rapid method for assessing the somatic energy content of migrating adult Pacific salmon. Trans Am Fish Soc 134: 184–191. 10.1577/FT04-076.1.

[ref12] Daigle NJ , Sacobie CFD, Verhille CE, Benfey TJ (2021) Triploidy affects postprandial ammonia excretion but not specific dynamic action in 1+ brook charr, *Salvelinus** fontinalis*. Aquaculture 536: 736503. 10.1016/j.aquaculture.2021.736503.

[ref13] Deslauriers D , Heironimus LB, Chipps SR (2016) Test of a foraging-bioenergetics model to evaluate growth dynamics of endangered pallid sturgeon (*Scaphirhynchus albus*). Ecol Model 336: 1–12. 10.1016/j.ecolmodel.2016.05.017.

[ref14] Distell (2011) User manual: Distell Fatmeter. Version 2.8. https://assurantinnovations.com/wp-content/uploads/2015/03/Fish-Fatmeter-User-Manual-v2.8.pdf. (last accessed 31 October 2022).

[ref15] Djokic MA , Heishman J, Kappenman KM, Gaylord TG, Verhille CE (2022) A microwave energy meter to estimate energetic reserves in juvenile sturgeon. J Appl Ichthyol 38: 149–156. 10.1111/jai.14311.

[ref16] Forbes SA , Richardson RE (1908) Order Chondrostei – the sturgeons. In: Forbes SA, Richardson RE, eds. *The Fishes of Illinois*. Authority of the State Legislature, Illinois Printing Company, Danville, pp. 21–29

[ref17] Fox J , Weisberg S, Price B (2021) Car: companion to applied regression. R Package Version 3.0-12. https://cran.r-project.org/web/packages/car/index.html. (last accessed 31 October 2022).

[ref18] Fuller DB , Jaeger M, Webb MAH (2007) *Spawning and Associated Movement Patterns of Pallid Sturgeon in the Lower Yellowstone** River**. *Report: Western Area Power Administration, Upper Basin Pallid Sturgeon Work Group, and U. S. Army Corps of Engineers, Bozeman, pp. 1–22

[ref19] Gerrity PC , Guy CS, Gardner WM (2006) Juvenile pallid sturgeon are piscivorous: a call for conserving native cyprinids. Trans Am Fish Soc 135: 604–609. 10.1577/T05-122.1.

[ref20] Grohs KL , Klumb RA, Chipps SR, Wanner GA (2009) Ontogenetic patterns in prey use by pallid sturgeon in the Missouri River, South Dakota and Nebraska. J Appl Ichthyol 25: 48–53. 10.1111/j.1439-0426.2009.01279.x.

[ref21] Haller LY , Hung SS, Lee S, Fadel JG, Lee JH, McEnroe M, Fangue NA (2015) Effect of nutritional status on the osmoregulation of green sturgeon (*Acipenser medirostris*). Physiol Biochem Zool 88: 22–42. 10.1086/679519. 25590591

[ref22] Hamel MJ , Pegg MA, Goforth RR, Phelps QE, Steffensen KD, Hammen JJ, Rugg ML (2014) Range-wide age and growth characteristics of shovelnose sturgeon from mark–recapture data: implications for conservation and management. Can J Fish Aquat Sci 72: 71–82.

[ref23] Hamel MJ , Spurgeon JJ, Steffensen KD, Pegg MA (2020) Uncovering unique plasticity in life history of an endangered centenarian fish. Sci Rep 10: 12866. 10.1038/s41598-020-69911-1. 32733007 PMC7393173

[ref24] Heckel JJ (1836) *Scaphirhynchus*, eine neue Fischgattung aus der Ordnung der Chondropterygier mit freien Kiemen. Ann Wiener Mus Naturgesch 1: 69–79.

[ref25] Hendry AP , Beall E (2004) Energy use in spawning Atlantic salmon. Ecol Freshw Fish 13: 185–196. 10.1111/j.1600-0633.2004.00045.x.

[ref26] Holmquist LM , Guy CS, Tews A, Webb MAH (2019) First maturity and spawning periodicity of hatchery‐origin pallid sturgeon in the upper Missouri River above Fort Peck Reservoir, Montana. Archive of fishery and marine research 35: 138–148. 10.1111/jai.13751.

[ref27] IUCN (2022) The IUCN red list of threatened species. Version 2021-3. https://www.iucnredlist.org. (last accessed 31 October 2022).

[ref28] Jordan GR , Heist EJ, Braaten PJ, DeLonay AJ, Hartfield P, Herzog DP, Kappenman KM, Webb MAH (2016) Status of knowledge of the pallid sturgeon (*Scaphirhynchus albus* Forbes and Richardson, 1905). J Appl Ichthyol 32: 191–207. 10.1111/jai.13239.

[ref29] Kaga TS , Sato S, Nagasawa T, Fukuwaka M, Nomura T, Urawa S (2009) Rapid estimation of lipid content of immature chum salmon in the ocean with a handheld microwave meter. NPAFC Doc 1208, Vancouver 1–8.

[ref30] Keenlyne KD , Maxwell SJ (1993) Length conversions and length-weight relations for pallid sturgeon. N Am J Fish Manag 13: 395–397. 10.1577/1548-8675(1993)013<0395:LCALWR>2.3.CO;2.

[ref31] Kooijman SALM (2009) *Dynamic Energy Budget Theory for Metabolic Organization*, Third Edition. Cambridge University Press, Cambridge, pp. 1–514, 10.1017/CBO9780511805400.

[ref32] Lee S , Haller LY, Fangue NA, Fadel JG, Hung SSO (2016) Effects of feeding rate on growth performance and nutrient partitioning of young-of-the-year white sturgeon (*Acipenser transmontanus*). Aquacult Nutr 22: 400–409. 10.1111/anu.12255.

[ref33] Mesa MG , Rose BP (2015) An assessment of morphometric indices, blood chemistry variables and an energy meter as indicators of the whole body lipid content in *Micropterus dolomieu*, *Sander vitreus* and *Ictalurus punctatus*. J Fish Biol 86: 755–764. 10.1111/jfb.12600. 25545237

[ref34] Meyer HA , Chipps SR, Graeb BDS, Klumb RA (2016) Growth, food consumption, and energy status of juvenile pallid sturgeon fed natural and artificial diets. J Fish Wildl Manag 7: 388–396. 10.3996/082015-JFWM-076.

[ref35] Powell MS , Hardy RW, Flagg TA, Kline PA (2010) Proximate composition and fatty acid differences in hatchery-reared and wild Snake River sockeye salmon overwintering in nursery lakes. N Am J Fish Manag 30: 530–537. 10.1577/M09-002.1.

[ref36] R Core Team (2023) The R Project for Statistical Computing. https://www.r-project.org. (last accessed 14 March 2023).

[ref37] Randall MT , Colvin ME, Steffensen KD, Welker TL, Pierce LL, Jacobson RB (2017) *Assessment of Adult Pallid Sturgeon Fish Condition, Lower Missouri River - Application of New Information to the Missouri River Recovery** Program*. U.S. Geological Survey Open-File Report 2017–1121, Reston, pp. 1–103

[ref38] Rosner B (1983) Percentage points for a generalized ESD many-outlier procedure. Dent Tech 25: 165–172. 10.1080/00401706.1983.10487848.

[ref39] Rotella J (2017) *Upper Basin Pallid Sturgeon Survival Estimation Project - 2017 Update. Upper Basin Workgroup Report**.* Montana State University, Bozeman, pp. 1–121

[ref40] Schloesser RW , Fabrizio MC (2017) Condition indices as surrogates of energy density and lipid content in juveniles of three fish species. Trans Am Fish Soc 146: 1058–1069. 10.1080/00028487.2017.1324523.

[ref41] Shuman DA , Klumb RA, Wilson RH, Jaeger ME, Haddix T, Gardner WM, Doyle WJ, Horner PT, Ruggles M, Steffensen KD et al. (2011) Pallid sturgeon size structure, condition, and growth in the Missouri River Basin. J Appl Ichthyol 27: 269–281. 10.1111/j.1439-0426.2010.01645.x.

[ref42] Steffensen KD (2018) Potential minimum threshold for pallid sturgeon relative condition in the lower Missouri River. Trans Nebr Acad Sci Affil Soc 38: 19–25.

[ref43] Steffensen KD , Hamel MJ, Spurgeon JJ (2019) Post-stocking pallid sturgeon *Scaphirhynchus albus* growth, dispersal, and survival in the lower Missouri River. J Appl Ichthyol 35: 117–127. 10.1111/jai.13646.

[ref44] Steffensen KD , Mestl GE (2016) Assessment of pallid sturgeon relative condition in the upper channelized Missouri River. J Freshwater Ecol 31: 583–595. 10.1080/02705060.2016.1196465.

[ref45] Upper Basin Pallid Sturgeon Propagation Committee (2005) Upper Basin Pallid Sturgeon Propagation Plan. Report: Upper Basin Pallid Sturgeon Propagation Committee, Billings, pp. 1–91

[ref46] USFWS (1990) Endangered and threatened wildlife and plants; determinations of endangered status for the pallid sturgeon. Fed Reg 55: 36641–36647.

[ref47] USFWS (2014) *Revised Pallid Sturgeon Scaphirhynchus albus Range-Wide Stocking Plan*. U.S. Fish and Wildlife Service Report, Denver, pp. 1–44

[ref48] USFWS (2019) *Revised Pallid Sturgeon Scaphirhynchus albus Range-Wide Stocking Plan*. U.S. Fish and Wildlife Service Report, Denver, pp. 1–55

[ref49] Velleman PF , Hoaglin DC (1981) Re-expression and the ladder of powers. In: Velleman PF, Hoaglin DC, eds. *Applications, Basics, and Computing of Exploratory Data** Analysis*. Duxbury Press, Boston, pp. 48–50

[ref50] Vogt A , Gormley R, Downey G, Somers J (2002) A comparison of selected rapid methods for fat measurement in fresh herring (*Clupea harengus*). J Food Compost Anal 15: 205–215. 10.1006/jfca.2002.1049.

[ref51] Webb MAH , Van Eenennaam JP, Crossman JA, Chapman FA (2019) A practical guide for assigning sex and stage of maturity in sturgeons and paddlefish. J Appl Ichthyol 35: 169–186. 10.1111/jai.13582.

[ref52] Wilson R , Hultberg S, Sandness Z (2017) *2016 Annual Report Pallid Sturgeon Population Assessment and Associated Fish Community Monitoring for the Missouri River: Segment 4**.* U.S. Fish and Wildlife Service Report, Bismarck, pp. 1–104

[ref53] Wuenschel MJ , McElroy WD, Oliveira K, McBride RS (2019) Measuring fish condition: an evaluation of new and old metrics for three species with contrasting life histories. Can J Fish Aquat Sci 76: 886–903. 10.1139/cjfas-2018-0076.

