# Supplementary material for: Validation of a microwave energy meter to non-lethally estimate energetic reserves in adult sturgeon
Source: Conserv Physiol. 2023 May 9;11(1):coad023. doi: 10.1093/conphys/coad023 (PMC10170255; doi:10.1093/conphys/coad023)
Supplement: Web_Material_coad023 [file web_material_coad023.zip › Supplementary_Material_Daigle_et_al_Mar_18.pdf]

Table S1. Summary of literature where commercially available microwave energy meters were tested as a method to estimate whole-body (WB) energetic reserves in various fish species. The data ranges were taken directly from the referenced publications or estimated from figures when data ranges were not directly reported. For comparison purposes, all data have been presented in percentage (%) for WB lipid and calories per gram (cal/g) for WB energy. A dash represents data that were not included or presented in the referenced publications.

Fish species	Fork length (FL; mm)	WB lipid (%)	R^2	WB energy (cal/g)	R^2	Reference(s)
Pacific salmon (*Oncorhynchus spp.*)	250-845	0-19.8	0.71-0.94	955-2627	0.65-0.93	Colt and Shearer, 2001; Crossin and Hinch, 2005; Kaga *et al.,* 2009
Atlantic croaker (*M. undulates*)	118-214 †	0.8-10.3	0.64-0.68	715-1800	0.58-0.62	Schloesser and Fabrizio, 2017
Striped bass (*M. saxatilis*)	117-193	1.3-7.8	0.07-0.22	822-1642	0.02-0.12	Schloesser and Fabrizio, 2017
Summer flounder (*P. dentatus*)	156-289 †	0.6-4.7	0.39-0.41	650-1269	0.33-0.44	Schloesser and Fabrizio, 2017
American shad (*A. sapidissima*)	-	1-12	0.72	-	-	Bayse *et al.,* 2018
Atlantic herring (*C. harengus*)	-	2-15	0.71	-	-	Vogt *et al.,* 2002
Smallmouth bass (*M. dolomieu*)	249-530	1-8	0.02	-	-	Mesa and Rose, 2015
Walleye (*S. viteus*)	321-707	3-15	0.17	-	-	Mesa and Rose, 2015
Channel catfish (*I. punctatus*)	179-580	5-24	0.83	-	-	Mesa and Rose, 2015
Atlantic salmon (*S. salar*)	-	0-25	0.30-0.87	-	-	Hendry and Beall, 2004
Pallid sturgeon (*S. albus*)	250-415	0-11.3	0-0.23	358-1930	0.01-0.21	Djokic *et al.,* 2022

Note: † symbolizes that total length (TL; mm) was used (and not FL).

Table S2. AICc rankings of competing regression models for estimating whole-body (WB) energetic reserves in captive adult pallid sturgeon, *Scaphirhynchus albus* (body mass [BM] 3342.7 ± 121.2g, mean ± SE), n = 43. Nine models were presented to include the top-ranked model considering only one Fatmeter site measurement (i.e., U-P); consistency was maintained throughout the table when possible. Abbreviations: AICc is second-order Akaike's Information Criteria; Kn is relative condition (based on Randall's (KnR) or Shuman's (KnS) FL-at-BM relationship); TL is total length; Avg denotes the average of the specified sites; the Fatmeter site codes define the position on the dorsoventral (U – upper and Ab - abdominal) and anteroposterior axes (A - anterior, M - middle, and P - posterior); *p* represents *p*-value, with * <0.05 and ** <0.001.

Model	WB Lipid (%)			WB Energy (kcal/100g)		
	AICc	*p*	R^2	AICc	*p*	R^2
		Body metrics only				
KnR (no units)	-84.8	**	0.49	-421.7	**	0.50
KnS (no units)	-82.8	**	0.47	-419.6	**	0.48
BM (g)	-81.4	**	0.45	-418.8	**	0.47
TL (mm)	-59.5	*	0.08	-395.7	*	0.09
		Fatmeter sites only				
Avg(Ab-P, U-A, U-P)	-109.6	**	0.71	-443.9	**	0.70
Avg(Ab-P, U-A, U-M, U-P)	-108.1	**	0.70	-444.7	**	0.71
Avg(Ab-P, U-P)	-107.9	**	0.70	-445.5	**	0.71
Avg(Ab-P, U-M, U-P)	-106.5	**	0.69	-446.2	**	0.72
Avg(Ab-P, U-A, U-M)	-104.0	**	0.67	-440.7	**	0.68
Avg(U-A, U-M, U-P)	-103.9	**	0.67	-439.5	**	0.67
Avg(U-A, U-P)	-103.5	**	0.67	-436.2	**	0.65
Avg(Ab-P, U-A)	-103.2	**	0.67	-437.3	**	0.66
U-P	-102.9	**	0.67	-438.6	**	0.67
		Combination of body metrics and Fatmeter sites				
Avg(Ab-P, U-A, U-P) + BM †	-115.0	**	0.76	-450.8	**	0.76
U-P + BM ‡ §	-114.5	**	0.75	-452.0	**	0.76
Avg(Ab-P, U-P) + BM	-112.0	**	0.74	-450.8	**	0.76
Avg(Ab-P, U-A, U-P) + TL	-108.4	**	0.72	-443.1	**	0.71
Avg(Ab-P, U-A, U-M) + KnR	-108.3	**	0.72	-445.8	**	0.73
Avg(Ab-P, U-A, U-M) + KnS	-107.5	**	0.71	-444.9	**	0.72
Avg(Ab-P, U-P) + TL	-106.1	**	0.70	-443.9	**	0.71
U-P + TL	-103.8	**	0.68	-440.0	**	0.69
Ab-P + KnR	-99.9	**	0.65	-438.5	**	0.68

† AICc top-ranked model for WB lipid; ‡ and energy; § Recommended model, determined based on the trade-off between AICc ranking and minimization of the number of Fatmeter measurements, to minimize fish handling time.

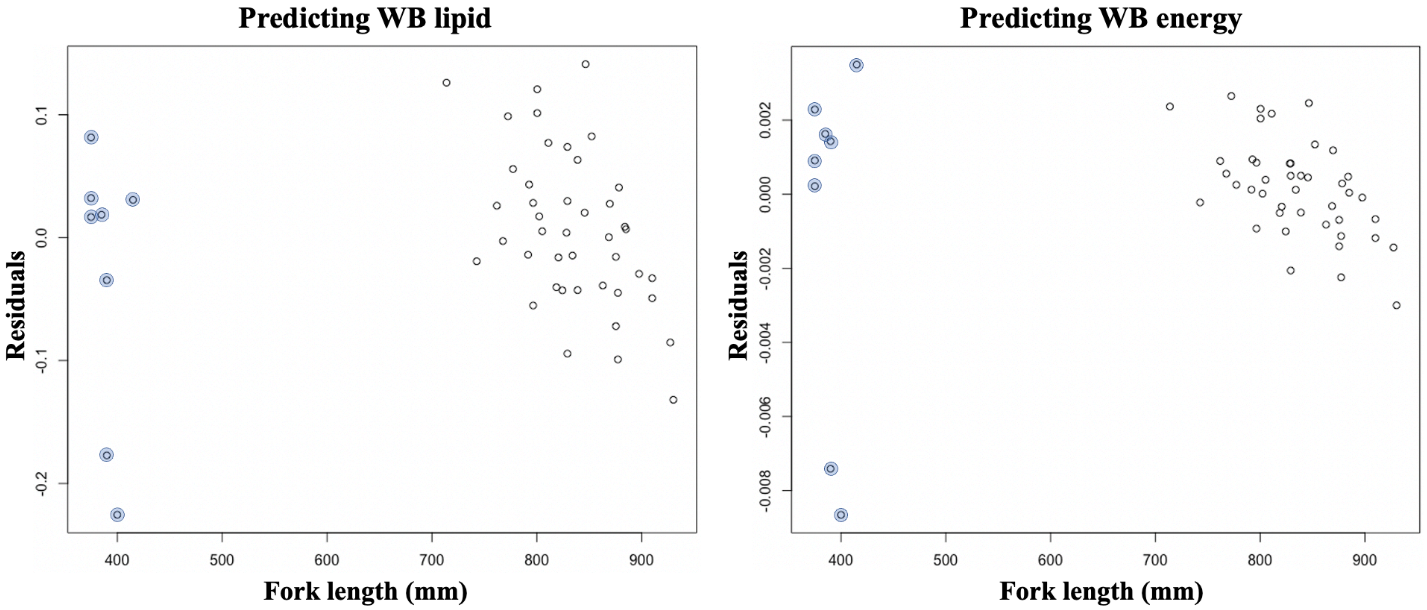

Figure S1. Residuals of estimates for pallid sturgeon, *Scaphirhynchus albus,* whole-body (WB) energetic reserves related to fork length using the recommended models (see Table 2 for recommended model formulae). Each data point represents the residuals for a single fish; the double circle points represent residuals for the largest (≥375mm fork length) juvenile pallid sturgeon from Djokic *et al.* (2022). The single circle points represent residuals for adult pallid sturgeon from the current study.
